# Exogenous Alpha-Ketoglutaric Acid Alleviates the Rabbit Dermal Papilla Cell Oxidative Damage Caused by Hydrogen Peroxide Through the ERK/Nrf2 Signaling Pathway

**DOI:** 10.3390/antiox14040455

**Published:** 2025-04-11

**Authors:** Xiaosong Wang, Shu Li, Jiali Chen, Lei Liu, Fuchang Li

**Affiliations:** Key Laboratory of Efficient Utilization of Non-Grain Feed Resources (Co-Construction by Ministry and Province), Ministry of Agriculture and Rural Affairs, Shandong Provincial Key Laboratory of Animal Nutrition and Efficient Feeding, Department of Animal Science and Technology, Shandong Agricultural University, Tai’an 271017, China; 14768123432@163.com (X.W.); 2021010083@sdau.edu.cn (S.L.); jlchen0723@163.com (J.C.)

**Keywords:** AKG, hydrogen peroxide (H_2_O_2_), oxidative stress, ERK/Nrf2, dermal papilla cells (DPCs)

## Abstract

As an endogenous metabolite, α-ketoglutarate (AKG) exhibits potent antioxidant properties, yet its molecular mechanisms remain unclear. Dermal Papilla Cells (DPCs), functioning as the regulatory hub of hair follicle morphogenesis, serve as a pivotal model system for deciphering follicular functionality and regeneration mechanisms through their orchestration of signaling networks. Using a hydrogen peroxide (H_2_O_2_)-induced oxidative stress model in DPCs, we investigated AKG’s protective effects. AKG attenuated H_2_O_2_-triggered reactive oxygen species (ROS) overproduction, restored mitochondrial membrane potential, and suppressed apoptosis-related protein dysregulation. It enhanced cellular stress resistance by increasing the Bcl-2/Bax ratio, boosting antioxidant levels, and inhibiting inflammation. Mechanistically, H_2_O_2_ activated the Nrf2 pathway, while AKG amplified Nrf2 nuclear translocation and expression. Crucially, ERK inhibition abrogated AKG-mediated Nrf2 regulation, intensifying ROS accumulation and cell death. These results identify the ERK/Nrf2 axis as central to AKG’s antioxidative cytoprotection. This study advances AKG’s therapeutic potential and deepens insights into its multifunctional roles.

## 1. Introduction

Oxidative stress refers to an imbalance between oxidants and antioxidants, with the equilibrium skewed toward oxidants, resulting in molecular damage and the disruption of redox signaling control [1]. Within eukaryotic cells, tripartite metabolic centers—mitochondria, the endoplasmic reticulum, and peroxisomes—demonstrate particular vulnerability to oxidative perturbations that disrupt cellular functionality. Such molecular disturbances propagate through biological hierarchies, ultimately manifesting as tissue dysfunction and systemic pathophysiological consequences [2,3]. In intensive livestock production systems, oxidative stress induces multifaceted detrimental outcomes including metabolic disorders, productivity impairment, reproductive failure, and elevated mortality rates, imposing significant economic burdens [4,5]. The domestic rabbit, as a typical burrowing species, exhibits heightened susceptibility to oxidative stress due to its diurnal activity rhythms and pronounced skittish behavioral characteristics. Domestic rabbits serve roles as economically valuable livestock species that are used as high-grade protein sources and premium fur products, and they are well-established biomedical models that contribute substantially to dermatological research [6]. Hair follicle units, as the principal cutaneous appendages, perform critical physiological functions including thermoregulation, environmental protection, and mechanosensation. Particularly in rabbits, follicular morphogenesis determines pelt quality parameters while serving as an exemplary experimental system for investigating alopecia pathogenesis and therapeutic interventions [7,8]. Oxidative stress in animals can be triggered by diverse factors, including improper transportation, weaning, hyperthermia, mycotoxin-contaminated feed, and environmental stressors. Studies indicate that oxidative damage disrupts the hair follicle growth cycle, leading to pathological hair loss, and directly reduces melanocyte populations within follicles, resulting in hair depigmentation [9,10]. H_2_O_2_-induced oxidative stress in DPCs has been shown to promote apoptosis, diminish DPCs’ capacity to regulate hair follicle development, accelerate epidermal differentiation, and inhibit the proliferation of hair follicle stem cells [11]. These findings underscore the profound detrimental effects of oxidative stress on fur-bearing animals and humans, extending to psychological distress and economic losses.

α-ketoglutarate (AKG), a pivotal intermediate in the tricarboxylic acid (TCA) cycle, plays an essential role in energy metabolism and amino acid biosynthesis. The intracellular functionality of AKG primarily relies on the glutamine–AKG axis, through which the bidirectional interconversion between glutamine and AKG is mediated by enzymatic activities including those of glutamate dehydrogenase, glutamine synthetase, glutamine transaminase, and ω-amidase [12]. As a stable precursor of glutamine, AKG has garnered increasing attention due to its stable physicochemical properties, cost-effectiveness, favorable aqueous solubility, and minimal toxicity. The antioxidant mechanisms of AKG are primarily attributed to two pathways. First, AKG enhances enzymatic antioxidant defense systems by upregulating key antioxidant components. For instance, AKG alleviates lipopolysaccharide-induced sepsis in piglet spleens by elevating catalase (CAT), total superoxide dismutase (T-SOD), glutathione (GSH), and glutathione peroxidase (GPX) levels [13]. Additionally, it mitigates cadmium-induced hepatocyte inflammation by reducing malondialdehyde (MDA) levels while increasing GSH and SOD activities [14]. Second, AKG directly interacts with H_2_O_2_ via non-enzymatic oxidative decarboxylation, generating succinate, water, and carbon dioxide [15]. Based on the cumulative evidence presented, we hypothesize that the robust antioxidant capacity of AKG may confer a potential protective effect on hair follicle integrity. However, the molecular mechanisms underlying AKG’s antioxidant capacity remain insufficiently elucidated. This study aims to investigate the molecular mechanisms by which AKG alleviates hydrogen peroxide-induced oxidative stress in DPCs, thereby providing foundational data for combating oxidative stress-related pathologies.

## 2. Materials and Methods

### 2.1. Reagents and Antibodies

Dimethyl α-ketoglutarate (DMKG, purity > 95%) was purchased from Glpbio (Montclair, CA, USA). Dulbecco’s Modified Eagle Medium (DMEM) and fetal bovine serum (FBS) were procured from Wuhan Pricella Biotechnology Co., Ltd. (Wuhan, China). Cell Counting Kit-8 (CCK-8), RIPA lysis buffer, and protease inhibitor cocktail were obtained from NCM Biotech (Suzhou, China). Nuclear and Cytoplasmic Protein Extraction Kit was sourced from Yeasen Biotechnology Co., Ltd. (Shanghai, China). MDA, T-SOD, T-AOC, oxidized glutathione (GSSG), and total glutathione (T-GSH) assay kits were acquired from Nanjing Jiancheng Bioengineering Institute (Nanjing, China). Bicinchoninic acid (BCA) protein assay kit was purchased from Kangwei Century Biotechnology Co., Ltd. (Taizhou, China). JNK inhibitor (SP600125), p38 inhibitor (SB203580), ERK1/2 inhibitor (U0126), and ROS detection kit were obtained from Beyotime Biotechnology (Shanghai, China). Mitochondrial function detection kit, RNAex Pro Reagent, Evo M-MLV reverse transcriptase kit, and SYBR Green Pro Taq HS Premix qPCR kit were provided by Accurate Biology Co., Ltd. (Changsha, China). Polyvinylidene fluoride (PVDF) membranes were procured from Monad Biotechnology Co., Ltd. (Suzhou, China).

Antibodies used in this study were sourced from multiple vendors: HRP-conjugated GAPDH monoclonal antibody was obtained from Proteintench (Wuhan, China). Antibodies against c-Jun N-terminal kinase (JNK), phosphorylated c-Jun N-terminal kinase (p-JNK), extracellular regulated protein kinases 1/2 (ERK1/2), phosphorylated extracellular regulated protein kinases 1/2 (p-ERK1/2), and p38 MAPK (p38) were purchased from Cell Signaling Technology (Danvers, MA, USA). Antibodies targeting phosphorylated p38 MAPK (p-p38), Nuclear factor erythroid 2-related factor 2 (Nrf2), phosphorylated Nuclear factor erythroid 2-related factor 2 (p-Nrf2), and proliferating cell nuclear antigen (PCNA) were acquired from Biosynthesis Biotechnology Co., Ltd. (Beijing, China). Caspase-3 (Cas3), caspase-8 (Cas8), B-cell lymphoma-2 (Bcl2), Bcl-2-associated X protein (Bax), interleukin-2 (IL-2), interleukin-6 (IL-6), interleukin-8 (IL-8), horseradish peroxidase (HRP)-conjugated goat anti-mouse IgG (H+L), HRP-conjugated goat anti-rabbit IgG (H+L), Western primary antibody dilution buffer, Western secondary antibody dilution buffer, and BeyoECL Plus chemiluminescence substrate were procured from Beyotime Biotechnology (Shanghai, China). All antibodies were diluted at ratio of 1:1000 as recommended by manufacturers.

### 2.2. Cell Culture and Experimental Design

The DPCs from Rex rabbit skin used in this study were provided by Prof. Xinsheng Wu at the College of Animal Science and Technology, Yangzhou University (Yangzhou, China). AKG, an organic weak acid, cannot passively traverse cell membranes; its hydrolyzable ester derivative DMKG was employed. DMKG readily crosses the plasma membrane and is enzymatically hydrolyzed intracellularly to release AKG [16]. DPCs, as mesenchymal cells, were cultured in DMEM supplemented with 10% FBS under standard conditions (37 °C, 5% CO_2_, and humidified atmosphere). This experiment was divided into four groups: a blank control group (C), an AKG pretreatment group (C+A), a H_2_O_2_ treatment group (H), and a H_2_O_2_ + AKG pretreatment group (H+A).

### 2.3. Cell Viability Assay

Cells were uniformly seeded in 96-well plates and allowed to reach an appropriate confluence prior to drug treatment. Following incubation, the drug-containing DMEM was aspirated, and cells were gently washed 2–3 times with phosphate-buffered saline (PBS). Subsequently, CCK-8 reagent was added to each well, and the plates were incubated at 37 °C for 2 h. The optical density (OD) was measured at 450 nm using a microplate reader (SpectraMax i3x, Molecular Devices, San Jose, CA, USA).

### 2.4. Determination of Antioxidant Enzyme Activity

The intracellular levels of MDA, T-SOD, total antioxidant capacity (T-AOC), GSSG, and T-GSH were measured using commercially available assay kits according to the manufacturer’s protocols. Total protein concentration was quantified using the BCA protein assay kit.

### 2.5. ROS Assay

Following the manufacturer’s protocol, treated cells were subjected to intracellular ROS detection using DCFH-DA (2′,7′-dichlorodihydrofluorescein diacetate). Briefly, the culture medium was aspirated from prepared cells, followed by incubation with 1 mL of DCFH-DA working solution (diluted in serum-free medium at a ratio specified by the kit) in a 37 °C cell culture incubator for 30 min. To eliminate the non-internalized probe, cells were rigorously washed three times with precooling serum-free medium under gentle agitation. Fluorescence microscopy analysis was immediately conducted using a fluorescence microscope (Nikon, Ts2R-FL, Minato City, Japan) equipped with an FITC filter set (excitation: 488 nm; emission: 525 nm). Image acquisition parameters were standardized across experimental groups, and a quantitative analysis of fluorescence intensity was performed using Image-J (1.54 g).

### 2.6. Mitochondrial Membrane Potential Assessment (Early Apoptosis Detection)

Mitochondrial membrane potential was evaluated using a JC-10 fluorescent probe according to standardized protocols. JC-10 working solution was freshly prepared as specified by the assay kit. Cultured cells in 6-well plates were washed twice with PBS to remove the residual medium. Subsequently, 1 mL of JC-10 working solution was applied to each well, and plates were gently rocked to ensure the complete coverage of the cellular monolayer. Cells were incubated at 37 °C under 5% CO₂ for 30 min to allow for JC-10 internalization and mitochondrial accumulation. Post-incubation, cells were rinsed twice with PBS to remove unbound dye. Fluorescence imaging was performed immediately using a fluorescence microscope (Nikon, Ts2R-FL, Japan) with dual-band filters for JC-10 detection (green monomeric form: excitation/emission 490/530 nm; red J-aggregate form: excitation/emission 540/590 nm). Image acquisition parameters were maintained uniformly across all experimental groups. For quantitative analysis, fluorescence intensity ratios (red–green) were calculated using Image-J (1.54 g). All procedures were conducted under low-light conditions to minimize photobleaching.

### 2.7. RNA Extraction and Quantification

Total RNA was isolated using RNAex Pro Reagent according to the following protocol: Cells in culture dishes were lysed with an appropriate volume of RNAex Pro Reagent and scraped into 1.5 mL microcentrifuge tubes. After homogenization, an equal volume of chloroform was added, and the mixture was vigorously vortexed for 30 s. Phase separation was achieved via centrifugation at 12,000× *g* for 15 min at 4 °C. The aqueous phase was transferred to a fresh tube, and RNA was precipitated by adding isopropanol, followed by centrifugation at 12,000× *g* for 15 min at 4 °C. The RNA pellet was washed twice with 75% ethanol, air-dried, and resuspended in RNase-free water. RNA concentration and purity were determined using an ultramicro-spectrophotometer. Reverse transcription was performed with an Evo M-MLV RT Kit, and quantitative PCR analysis was conducted using the SYBR Green Pro Taq HS Premixed qPCR Kit. GAPDH served as the endogenous control for normalization. Relative gene expression levels were calculated using the comparative threshold cycle (2^−ΔΔCT^) method. Primer sequences are provided in Table 1.

### 2.8. Western Blotting

Cellular proteins were extracted by adding an appropriate volume of ice-cold RIPA lysis buffer supplemented with a protease inhibitor cocktail to the culture dish. Cells were detached using a cell scraper and transferred to a 1.5 mL microcentrifuge tube. Following complete cell lysis, the lysate was centrifuged at 12,000× *g* for 10 min at 4 °C. The supernatant was collected, and protein concentration was quantified using the BCA protein assay kit. For immunoblotting, protein samples were denatured and subjected to SDS-PAGE. Subsequently, resolved proteins were electrophoretically transferred onto a 0.45 μm PVDF membrane. The membrane was blocked with rapid blocking buffer for 1 h at room temperature, followed by three 5 min washes with Tris–Borate–Sodium Tween-20 (TBST). Primary antibodies were applied and incubated overnight at 4 °C. After additional TBST washes, horseradish peroxidase (HRP)-conjugated secondary antibodies were incubated with the membrane for 1–2 h at room temperature. Protein bands were visualized using a chemiluminescence detection system (Vilber Bio Imaging, Paris, France), and densitometric analysis was performed to quantify band intensity.

### 2.9. Data Statistics and Analysis

The experimental data were initially processed and organized using Microsoft Excel. Comparative statistical analyses were performed with SAS 9.4 (SAS Institute Inc., Cary, NC, USA), and graphical representations were generated using GraphPad Prism 9 (GraphPad Software, San Diego, CA, USA). A threshold of *p* < 0.05 was applied to determine statistical significance, while *p* > 0.05 indicated non-significance. All datasets were derived from a minimum of three independent experimental replicates and are presented as the mean ± standard deviation (SD).

## 3. Results

### 3.1. AKG Alleviated H_2_O_2_-Induced Decrease in Cell Viability

**Figure 1:**
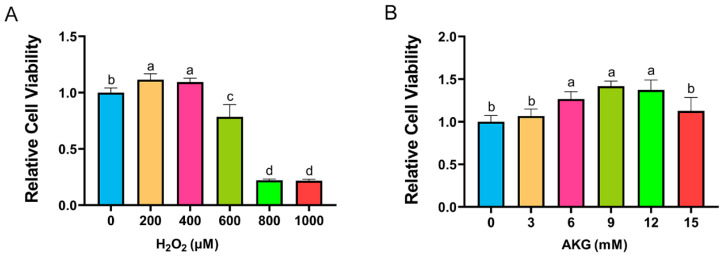
AKG alleviated H_2_O_2_-induced decrease in cell viability. (**A**) Changes in cell viability following 12 h exposure to different concentrations of H_2_O_2_. (**B**) Effects of 24 h AKG pretreatment followed by 12 h 600 μm H_2_O_2_ exposure on cell viability. Data are presented as mean ± SD (n = 12). Different letters indicate statistically significant differences (*p* < 0.05), while shared letters denote no significant differences (*p* > 0.05).

Cell viability, a critical indicator of cellular health, was quantified using a CCK-8 assay. According to the method of Sheng et al., DPCs were exposed to H_2_O_2_ at a concentration of 0, 200, 400, 600, 800, or 1000 μM for 12 h [17]. As shown in Figure 1A, H_2_O_2_ at 200 and 400 μM unexpectedly enhanced cell viability. However, a marked reduction in viability was observed at 600 μM H_2_O_2_, with survival rates plummeting to 20% at 800–1000 μM. Consequently, 600 μM H_2_O_2_ treatment for 12 h was selected to establish the oxidative stress model. To investigate the protective role of AKG, cells were pretreated with varying AKG concentrations (3–15 mM) for 24 h, followed by 600 μM H_2_O_2_ exposure for 12 h, as adapted from Cheng et al. [18]. The CCK-8 assay revealed that 6, 9, and 12 mM AKG significantly restored viability in oxidatively stressed cells, whereas 3 and 15 mM AKG showed no statistically significant effects (Figure 1B). Based on these findings, the subsequent experiments employed 6 mM AKG pretreatment for 24 h prior to the H_2_O_2_ challenge.

### 3.2. AKG Reduced ROS Production

**Figure 2:**
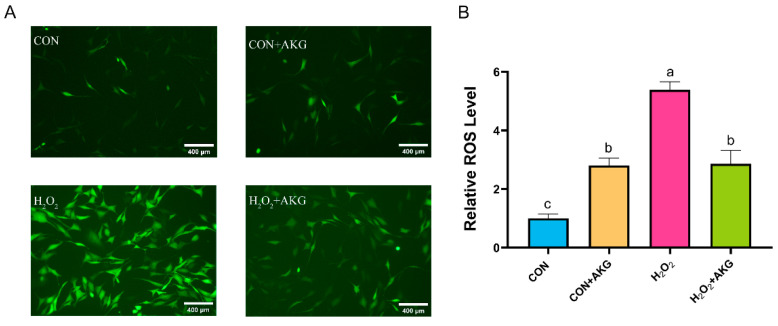
AKG reduced the H_2_O_2_-induced increase in ROS. DPCs were pretreated with 6 mM AKG for 24 h and then exposed to 600 μM H_2_O_2_ for 12 h. (**A**) Intracellular ROS content under different treatments. Scale bar = 400 µm. (**B**) Data visualization and statistical analysis. The fluorescent dye used in this assay was the DCFH-DA probe. Data are presented as the mean ± SD (n = 3). Different letters indicate statistically significant differences (*p* < 0.05), while shared letters denote no significant differences (*p* > 0.05).

ROS play critical roles in cellular metabolism, but excessive ROS accumulation can lead to intracellular DNA damage, inflammation, and even cell death. Therefore, we first measured intracellular ROS levels following H_2_O_2_ treatment (Figure 2). The results demonstrated that H_2_O_2_ treatment significantly elevated intracellular ROS levels. Compared to the H_2_O_2_-treated group, AKG pretreatment markedly reduced ROS accumulation. Notably, AKG pretreatment alone also increased intracellular ROS levels.

### 3.3. AKG Restores Mitochondrial Membrane Potential Impaired by Oxidative Stress

**Figure 3:**
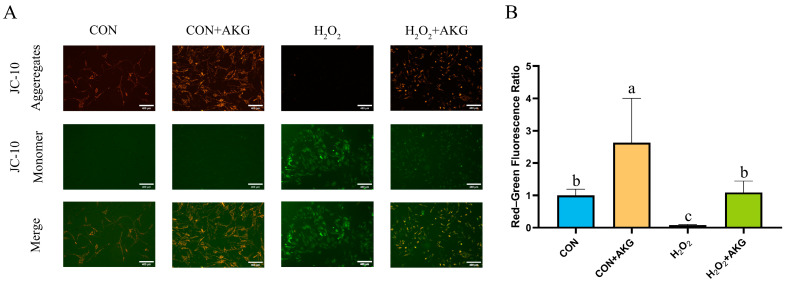
AKG alleviates H_2_O_2_-induced decline in mitochondrial membrane potential. DPCs were pretreated with 6 mM AKG for 24 h and then exposed to 600 μM H_2_O_2_ for 12 h. (**A**) Intracellular mitochondrial membrane potential under different treatments. Scale bar = 400 µm. (**B**) Data visualization and statistical analysis. Fluorescent dye used in this assay was JC-10 probe. Data visualization and statistical analysis. Data are presented as mean ± SD (n = 3). Different letters indicate statistically significant differences (*p* < 0.05), while shared letters denote no significant differences (*p* > 0.05).

A decline in mitochondrial membrane potential is recognized as a hallmark of early apoptosis. To investigate the effects of H_2_O_2_ treatment and AKG pretreatment on early apoptosis, we assessed changes in mitochondrial membrane potential (Figure 3). The results revealed that H_2_O_2_ treatment significantly reduced mitochondrial membrane potential, thereby promoting early apoptosis. In contrast, AKG pretreatment completely reversed this trend, restoring the proportion of early apoptotic cells to normal levels.

### 3.4. AKG Enhances Cellular Resistance to Apoptosis

**Figure 4:**
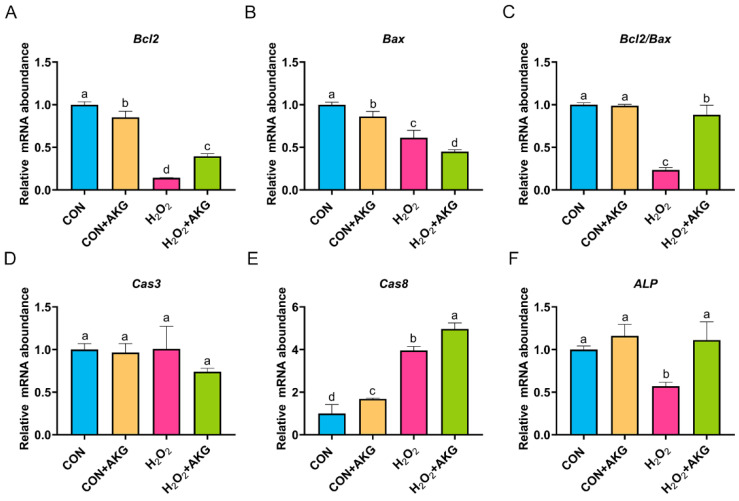
AKG enhances intracellular anti-apoptotic gene expression. DPCs were pretreated with 6 mM AKG for 24 h and then exposed to 600 μM H_2_O_2_ for 12 h. (**A**) *Bcl2* mRNA abundance; (**B**) *Bax* mRNA abundance; (**C**) *Bcl2/Bax* mRNA abundance; (**D**) *Cas3* mRNA abundance; (**E**) *Cas8* mRNA abundance; (**F**) *ALP* mRNA abundance. Data are presented as mean ± SD (n = 3). Different letters indicate statistically significant differences (*p* < 0.05), while shared letters denote no significant differences (*p* > 0.05).

In Section 3.3, we demonstrated that AKG effectively attenuates the early apoptotic propensity of DPCs. To mechanistically validate this observation, we performed quantitative real-time PCR analysis to determine the relative expression levels of critical anti-apoptotic and pro-apoptotic regulatory genes. As depicted in Figure 4, at the level of apoptosis-related gene expression, H_2_O_2_ treatment significantly reduced the expression of both *Bcl2* and *Bax*. Notably, the AKG pretreatment group exhibited a differential regulatory effect: *Bcl2* expression showed a statistically significant restorative upregulation compared to the H_2_O_2_ group (*p* < 0.05), while *Bax* expression further decreased. The *Bcl2/Bax* ratio, a critical indicator of apoptotic susceptibility, was significantly reduced in the H_2_O_2_ group. However, AKG pretreatment effectively reversed this trend, restoring the *Bcl2/Bax* ratio to near-normal levels. It is noteworthy that we observed a similar expression pattern in *alkaline phosphatase* (*ALP*), where H_2_O_2_ treatment suppressed *ALP* levels, while AKG supplementation reversed this trend. Following H_2_O_2_ exposure, the expression of *Cas3* remained comparable to untreated controls, whereas AKG treatment significantly downregulated *Cas3* transcription. Notably, H_2_O_2_ administration markedly elevated *Cas8* expression relative to baseline levels; however, this effect was paradoxically exacerbated upon AKG cotreatment, a phenomenon warranting further mechanistic investigation.

### 3.5. AKG Downregulates Expression of Inflammatory Factors and Apoptotic Proteins

**Figure 5:**
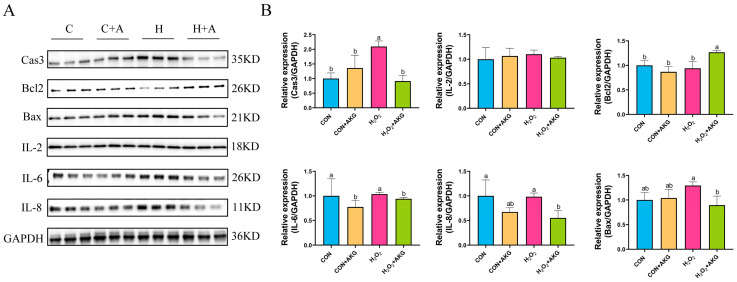
AKG downregulates intracellular inflammatory factors and upregulates anti-apoptotic protein expression. DPCs were pretreated with 6 mM AKG for 24 h and then exposed to 600 μM H_2_O_2_ for 12 h. (**A**) Protein expression. (**B**) Data visualization and statistical analysis. Data are presented as mean ± SD (n = 3). Different letters indicate statistically significant differences (*p* < 0.05), while shared letters denote no significant differences (*p* > 0.05).

The occurrence of intracellular oxidative stress is typically closely associated with cellular apoptosis and the upregulation of inflammatory factors. To comprehensively investigate the protective effects of AKG against oxidative stress in cells, we systematically examined the expression levels of apoptosis-related proteins, anti-apoptotic proteins, and inflammatory factors. As shown in Figure 5, H_2_O_2_ treatment significantly elevated intracellular Cas3 protein expression while showing no significant effects on IL-8, IL-6, Bcl2, or Bax expression. However, compared to the H_2_O_2_ group, AKG pretreatment remarkably reduced the intracellular levels of IL-8, IL-6, Bax, and Cas3 proteins while significantly upregulating Bcl2 protein expression. Unexpectedly, IL-2 expression remained unaffected by either H_2_O_2_ or AKG treatment.

### 3.6. AKG Enhances Cellular Antioxidant Capacity

**Figure 6:**
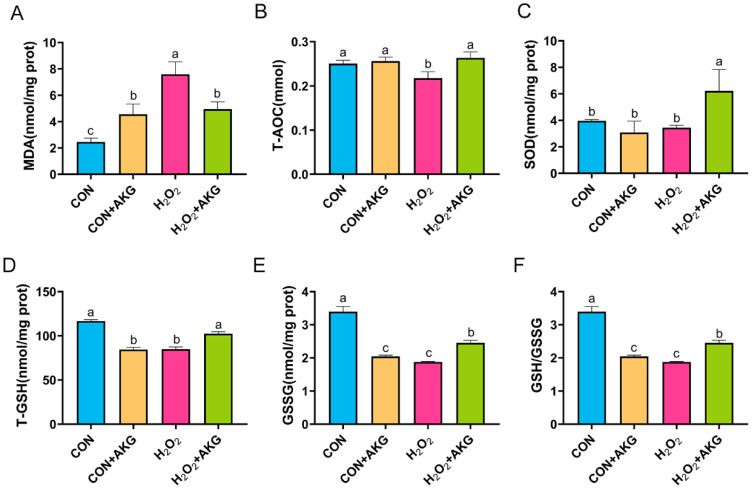
AKG enhances the activity of intracellular antioxidant enzymes. DPCs were pretreated with 6 mM AKG for 24 h and then exposed to 600 μM H_2_O_2_ for 12 h. (**A**) MDA content; (**B**) T-AOC content; (**C**) SOD content; (**D**) T-GSH content; (**E**) GSSG content; (**F**) GSH/GSSG ratio. Data are presented as the mean ± SD (n = 3). Different letters indicate statistically significant differences (*p* < 0.05), while shared letters denote no significant differences (*p* > 0.05).

Intracellular antioxidant enzymes constitute critical defense mechanisms against oxidative stress, with their activity levels directly reflecting cellular antioxidant capacity. To determine whether AKG-mediated cytoprotection against oxidative damage involves the modulation of these enzymes, we quantitatively assessed key antioxidant enzymatic profiles (Figure 6). Our results showed that compared to the control group, H_2_O_2_ treatment significantly increased MDA levels, which were partially normalized by AKG pretreatment though remaining elevated relative to baseline. H_2_O_2_ exposure markedly reduced T-AOC, T-GSH, GSSG, and the glutathione redox ratio (GSH/GSSG), all of which were significantly restored by AKG pretreatment. Notably, while SOD activity remained unchanged following the H_2_O_2_ challenge, AKG pretreatment resulted in significantly elevated SOD levels compared to both the H_2_O_2_-treated and control groups.

### 3.7. H_2_O_2_ Activates the Nrf2 Signaling Pathway

**Figure 7:**
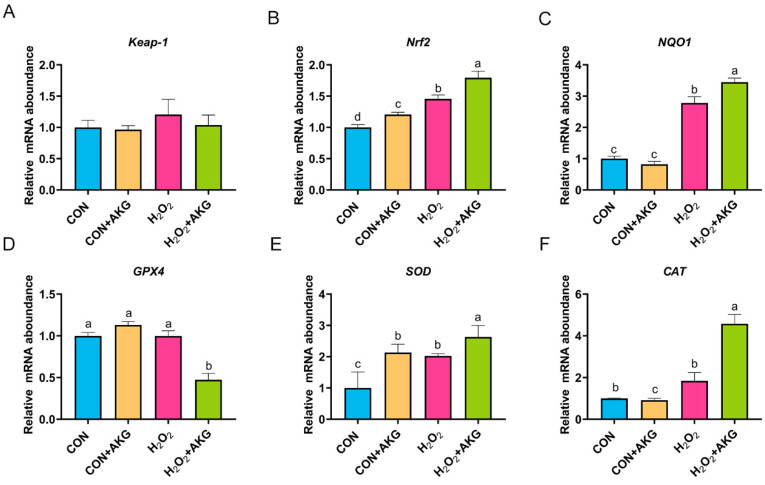
H_2_O_2_ upregulates the expression of Nrf2 signaling pathway-related genes. DPCs were pretreated with 6 mM AKG for 24 h and then exposed to 600 μM H_2_O_2_ for 12 h. (**A**) *Keap-1* mRNA abundance; (**B**) *Nrf2* mRNA abondance; (**C**) *NQO1* mRNA abundance; (**D**) *GPX4* mRNA abundance; (**E**) *SOD* mRNA abundance; (**F**) *CAT* mRNA abundance. Data are presented as the mean ± SD (n = 3). Different letters indicate statistically significant differences (*p* < 0.05), while shared letters denote no significant differences (*p* > 0.05).

Based on the aforementioned experimental results, we can conclude that AKG pretreatment effectively alleviates hydrogen peroxide-induced oxidative damage. However, the molecular mechanisms underlying the AKG-induced cytoprotective effects remain to be fully elucidated. The Nrf2 signaling pathway is recognized as the primary antioxidative defense mechanism. As illustrated in Figure 7, we measured the expression of key Nrf2 signaling pathway genes and downstream effector genes under oxidative stress and AKG pretreatment conditions. Subsequently, we analyzed the expression of Nrf2 pathway-related genes. Compared to the control group, the H_2_O_2_-treated group showed a non-significant upward trend in *Keap-1* expression, while *Nrf2* and *NQO1* expression levels were significantly elevated. The AKG pretreatment group demonstrated more pronounced regulatory effects than the H_2_O_2_ group, with the mRNA levels of *Nrf2* and *NQO1* increasing by 23.11% and 23.77%, respectively. These results suggest that H_2_O_2_ may activate the Nrf2 signaling pathway, and AKG enhances this activation. Finally, we evaluated downstream effectors. Compared to the control group, H_2_O_2_ treatment had no significant impact on *GPX4* or *CAT* expression. However, AKG pretreatment significantly reduced *GPX4* expression while increasing *CAT* expression by 4.6-fold. Additionally, H_2_O_2_ treatment significantly elevated *SOD* expression compared to the control, and AKG pretreatment further amplified this trend.

### 3.8. AKG Regulates Nrf2 Expression

**Figure 8:**
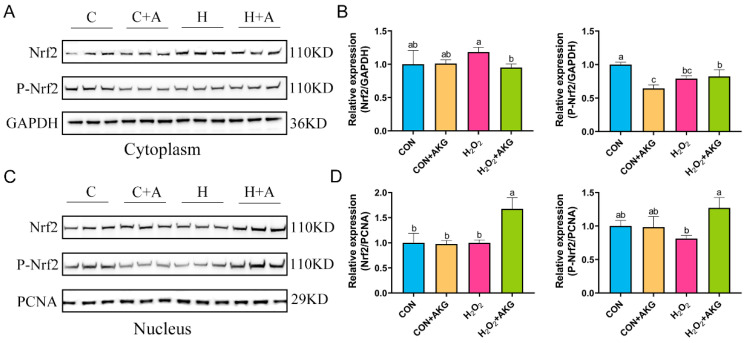
AKG increases nuclear Nrf2 protein expression. DPCs were pretreated with 6 mM AKG for 24 h and then exposed to 600 μM H_2_O_2_ for 12 h. (**A**) Nuclear Nrf2 protein expression. (**B**) Data visualization and statistical analysis. (**C**) Cytoplasmic Nrf2 protein expression. (**D**) Data visualization and statistical analysis. Data are presented as mean ± SD (n = 3). Different letters indicate statistically significant differences (*p* < 0.05), while shared letters denote no significant differences (*p* > 0.05).

The real-time quantitative PCR results suggested that H_2_O_2_ may activate the Nrf2 signaling pathway, and AKG pretreatment significantly augmented this activation trend. To further elucidate the regulatory mechanism of AKG in the Nrf2 signaling pathway, Western blot analysis was performed to determine the expression levels of the Nrf2 protein in the cytoplasmic and nuclear fractions (Figure 8). The results revealed that, compared with the H_2_O_2_ group, AKG pretreatment significantly reduced cytoplasmic Nrf2 expression, while no significant difference was observed in P-Nrf2 levels. Similarly, nuclear protein extraction demonstrated that AKG pretreatment markedly increased nuclear Nrf2 expression compared to other groups. Notably, P-Nrf2 levels in the AKG-pretreated group were significantly higher than those in the H_2_O_2_ group. These findings collectively suggest that AKG may enhance the antioxidant capacity of DPCs by modulating the Nrf2 signaling pathway.

### 3.9. H_2_O_2_ and AKG Perturb MAPK Signaling Pathway Dynamics

**Figure 9:**
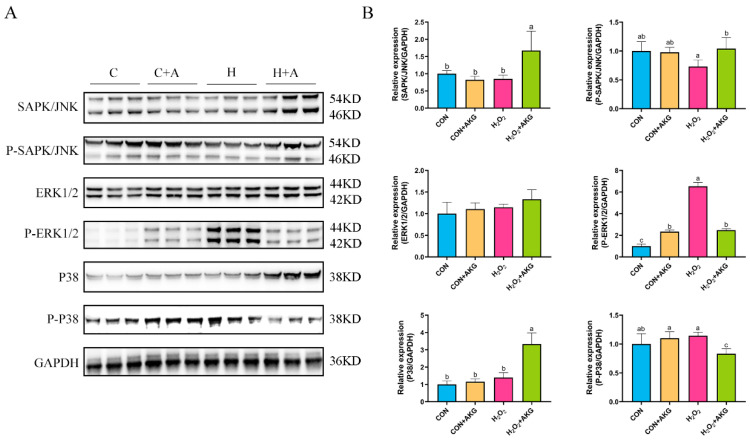
Effects of AKG and H_2_O_2_ on MAPK signaling pathway. DPCs were pretreated with 6 mM AKG for 24 h and then exposed to 600 μM H_2_O_2_ for 12 h. (**A**) Protein expression. (**B**) Data visualization and statistical analysis. Data are presented as mean ± SD (n = 3). Different letters indicate statistically significant differences (*p* < 0.05), while shared letters denote no significant differences (*p* > 0.05).

In Section 3.5, we observed that the Bcl2/Bax ratio was significantly elevated under AKG treatment, while the expression level of the Csa3 protein exhibited a marked reduction. However, these anti-apoptotic molecules are recognized as downstream effectors of the MAPK signaling pathway. We postulated that AKG may modulate alterations in the MAPK signaling pathway, thereby triggering a cascade of downstream molecular events. To validate this hypothesis, we investigated MAPK protein expression under oxidative stress conditions and examined the effects of AKG pretreatment. As shown in Figure 9, hydrogen peroxide treatment demonstrated no significant effects on JNK, P-JNK, ERK1/2, P38, or P-P38 protein levels compared to the control group. However, AKG pretreatment significantly upregulated the expression of the JNK and P-JNK1/2 proteins in oxidative stress-induced cells compared to the hydrogen peroxide-treated group. Although hydrogen peroxide treatment activated P-ERK1/2, this activation trend was significantly altered by AKG pretreatment. Notably, AKG pretreatment markedly increased P38 protein expression while simultaneously reducing P-P38 levels in oxidative stress conditions compared to other experimental groups.

### 3.10. ERK1/2 Regulates Nrf2 Protein Expression

**Figure 10:**
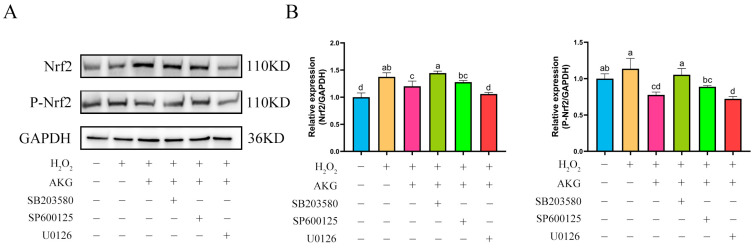
ERK1/2 inhibitor reduces Nrf2 protein expression. DPCs were pretreated with 6 mM AKG for 24 h and then exposed to 600 μM H_2_O_2_ for 12 h. (**A**) Protein expression. (**B**) Data visualization and statistical analysis. Data are presented as mean ± SD (n = 3). Different letters indicate statistically significant differences (*p* < 0.05), while shared letters denote no significant differences (*p* > 0.05).

The findings from Section 3.8 and Section 3.9 demonstrated that AKG pretreatment exerted significant regulatory effects on the expression of key proteins in both the Nrf2 and MAPK signaling pathways. Based on these findings, we hypothesized a potential regulatory relationship between the Nrf2 and MAPK pathways. To elucidate the specific regulatory mechanisms linking Nrf2 to the JNK, ERK1/2, and P38 proteins within the MAPK pathway, we conducted intervention experiments using specific inhibitors: JNK inhibitor SP600125, ERK1/2 inhibitor U0126, and P38 inhibitor SB203580. Changes in Nrf2 protein expression across experimental groups were analyzed via Western blot (Figure 10). Compared to the control group, AKG pretreatment significantly upregulated intracellular Nrf2 protein expression. Notably, the addition of the JNK or P38 inhibitor did not affect the AKG-induced upregulation of Nrf2 protein expression. However, when AKG pretreatment was combined with the ERK1/2 inhibitor, Nrf2 protein levels returned to baseline. Similar trends were observed for P-Nrf2: hydrogen peroxide treatment significantly reduced P-Nrf2 expression, and this reduction was further exacerbated by ERK1/2 inhibition.

### 3.11. ERK1/2 Regulates ROS Generation

**Figure 11:**
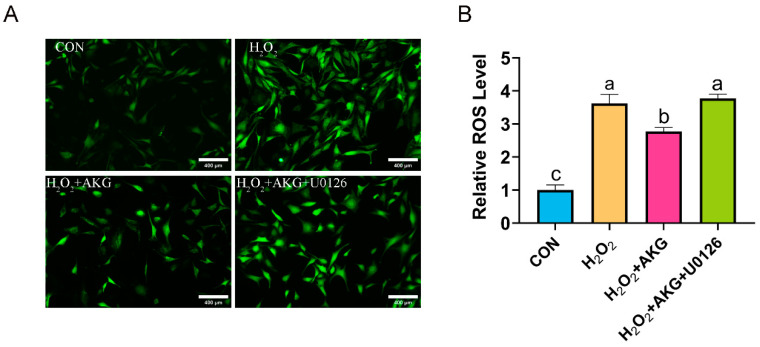
ERK1/2 inhibitor increases ROS generation. DPCs were pretreated with 6 mM AKG for 24 h and then exposed to 600 μM H_2_O_2_ for 12 h. (**A**) Intracellular ROS content under different treatments. Scale bar = 400 µm. (**B**) Data visualization and statistical analysis. Fluorescent dye used in this assay was DCFH-DA probe. Data are presented as mean ± SD (n = 3). Different letters indicate statistically significant differences (*p* < 0.05), while shared letters denote no significant differences (*p* > 0.05).

The aforementioned findings suggest that AKG may enhance cellular antioxidant capacity by modulating ERK1/2 protein expression to influence the Nrf2 signaling pathway. To further validate this speculation, we measured ROS levels under combined AKG pretreatment and ERK1/2-specific inhibitor intervention using the same methodology described in Section 3.2 (Figure 11). H_2_O_2_ treatment significantly elevated intracellular ROS levels compared to the control group. AKG pretreatment markedly attenuated this increase, though ROS levels remained significantly higher than those in the control. Strikingly, cotreatment with AKG and the ERK1/2 inhibitor restored ROS levels to those observed in the H_2_O_2_-treated group.

### 3.12. ERK1/2 Inhibitor Reduced Cell Viability

**Figure 12:**
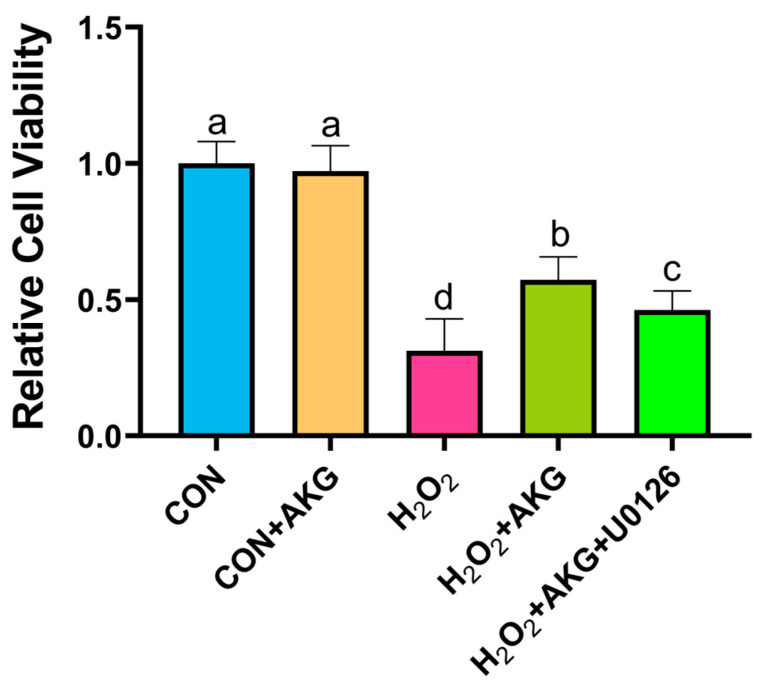
ERK1/2 inhibitor attenuates AKG-induced increase in cellular viability under oxidative stress. DPCs were pretreated with 6 mM AKG for 24 h and then exposed to 600 μM H_2_O_2_ for 12 h. Data are presented as mean ± SD (n = 12). Different letters indicate statistically significant differences (*p* < 0.05), while shared letters denote no significant differences (*p* > 0.05).

Using the methodology detailed in Section 3.1, we evaluated the impact of AKG pretreatment combined with ERK inhibition on cellular viability. As illustrated in Figure 12, H_2_O_2_ treatment significantly decreased cell viability compared to the control group. AKG pretreatment substantially restored viability in oxidative stress-induced cells. However, the co-administration of the ERK1/2 inhibitor U0126 with AKG pretreatment markedly reduced viability compared to AKG pretreatment alone. Notably, AKG pretreatment alone exhibited no statistically significant effect on viability relative to the untreated control.

## 4. Discussion

In cellular biology research, H_2_O_2_, as the most widely utilized oxidative stress inducer, primarily exerts its effects through the Fenton reaction with Fe^2+^, generating ROS that induce oxidative cellular damage [19]. However, the biological impacts of H_2_O_2_ are not exclusively detrimental. Previous studies have demonstrated that at specific concentrations, H_2_O_2_ may act as a pro-proliferative factor by promoting cell proliferation, with its metabolic byproduct ROS serving as an essential mediator for the G1-to-S phase transition [20,21]. The intracellular concentration of H_2_O_2_ is critically influenced by endogenous antioxidant defense systems. Notably, the rapid catalytic decomposition of exogenous H_2_O_2_ into water and oxygen by intracellular catalase has been documented when external H_2_O_2_ concentrations surge abruptly [22]. This efficient clearance mechanism results in significantly lower intracellular H_2_O_2_ levels compared to externally applied concentrations, providing a pivotal explanation for the concentration-dependent effects observed in this study: At low concentrations (200 and 400 μM), H_2_O_2_ enhanced cell viability by activating pro-proliferative signaling pathways, elevating intracellular oxygen levels, and augmenting metabolic rates. Conversely, the critical concentration of 600 μM exceeded the threshold of cellular antioxidant defenses, triggering uncontrolled ROS accumulation and the subsequent collapse of cell viability. Intriguingly, pretreatment with AKG for 24 h prior to 12 h exposure to 600 μM H_2_O_2_ significantly improved cell survival rates. This observation raises a pivotal scientific inquiry: what is the mechanism by which AKG restores the cell viability of DPCs under oxidative stress conditions?

Under physiological conditions, basal ROS production occurs as a natural byproduct of cellular metabolism. However, when ROS generation surpasses the scavenging capacity of antioxidant systems, oxidative stress ensues, initiating lipid peroxidation, protein denaturation, and DNA damage—pathological cascades that may ultimately lead to apoptosis [23]. Emerging evidence indicates that AKG mitigates reactive oxygen species (ROS) generation by facilitating the mitophagy-mediated clearance of dysfunctional mitochondria [24]. This phenomenon was further corroborated in our study through fluorescent probe analysis, which demonstrated that H_2_O_2_ treatment markedly elevated intracellular ROS levels, whereas AKG preconditioning substantially attenuated ROS accumulation, suggesting the AKG-mediated alleviation of oxidative stress and reduced burden on endogenous antioxidant systems. Intriguingly, in contrast to untreated controls, AKG monotherapy paradoxically induced a significant elevation in ROS levels. Mechanistically, prior studies have revealed AKG’s capacity to enhance mitochondrial membrane potential and ATP synthesis through the transcriptional regulation of mitochondrial complex I-associated genes in oocytes, thereby optimizing mitochondrial functionality [25]. We speculate that this transient ROS elevation observed under AKG treatment may stem from its dual regulatory effects: (1) augmented mitochondrial metabolic flux through tricarboxylic acid (TCA) cycle activation and (2) enhanced electron transport chain (ETC) activity, which generates ROS as an inevitable byproduct during transient metabolic adaptation. Our experimental data revealed that AKG treatment alone significantly elevated MDA levels and reduced the GSH/GSSG ratio in DPCs. Specifically, AKG supplementation likely enhances mitochondrial respiratory activity, thereby accelerating ETC flux and transiently elevating ROS production. Persistent ROS accumulation drives lipid peroxidation cascades, culminating in elevated MDA levels [26]. Concomitantly, the depletion of reduced GSH through ROS scavenging or the compensatory glutathione peroxidase-mediated detoxification of lipid hydroperoxides could mechanistically explain the observed decline in the GSH/GSSG ratio.

Subsequently, to further elucidate the protective effects of AKG, we investigated mitochondrial membrane potential and apoptosis-related molecular markers. A reduction in mitochondrial membrane potential has been recognized as a critical indicator of early-stage apoptosis [27]. The members of the Bcl-2 protein family serve as pivotal regulators in the mitochondrial-dependent apoptotic pathway, modulating apoptosis initiation or suppression through their interactions. Notably, Bax, a pro-apoptotic member of this family, undergoes activation and oligomerization at the mitochondrial outer membrane under apoptotic stimulation, thereby mediating alterations in mitochondrial membrane permeability—a decisive event in apoptotic progression. This process subsequently triggers an orderly cascade of signaling events culminating in apoptosis, with the caspase protease cascade system playing a central role in apoptosis signal induction, transduction, and amplification [28,29,30]. Consistent with previous reports demonstrating H_2_O_2_-induced apoptosis through Bcl-2 downregulation and Bax upregulation [31], our experimental data revealed parallel alterations at both the gene and protein levels. The observed decline in the Bcl-2/Bax ratio strongly indicates substantial apoptotic activation following H_2_O_2_ exposure. Remarkably, AKG pretreatment effectively reversed this pathological pattern by upregulating Bcl-2 expression while concurrently suppressing Bax levels, thereby demonstrating that AKG may potentially attenuate hydrogen peroxide-induced apoptosis by modulating the expression of Bcl-2 and Bax. As the initiator of caspase, Cas8 triggers proteolytic signaling during apoptosis, while Cas3 functions as the executioner protease responsible for terminal apoptotic events [32]. In our study, H_2_O_2_ exposure markedly elevated Cas3 protein levels, a pathological alteration effectively mitigated by AKG supplementation. These findings align with prior reports demonstrating H_2_O_2_-induced Cas3 upregulation in PC12 cells [33]. However, transcriptional analysis revealed no significant changes in *Cas3* mRNA expression, whereas *Cas8* gene expression peaked following AKG treatment. This discrepancy suggests that AKG exerts post-transcriptional regulation effects on Cas3, potentially modulating its translation or protein stability. Notably, Cas8 exhibits functional pleiotropy: beyond its canonical role in apoptosis initiation, emerging evidence implicates *Cas8* in necroptosis suppression via RIPK3-MLKL pathway modulation and immune homeostasis maintenance [34,35]. The AKG-induced *Cas8* upregulation observed herein may represent a dual regulatory mechanism, attenuating both apoptotic and necroptotic pathways to enhance cellular survival under oxidative stress.

In the context of inflammatory responses, IL-6, IL-8, and IL-2 represent the most extensively studied cytokines that play pivotal roles in inflammatory processes. IL-6 is transiently upregulated during the early phases of infection and tissue injury, facilitating host defense mechanisms through the stimulation of acute phase responses, hematopoiesis, and immune activation. As a key chemokine, IL-8 primarily mediates neutrophil recruitment and activation while regulating the directional migration of immune cells. IL-2 predominantly participates in immune cell activation and proliferation, modulating the functional states of immunocytes to initiate inflammatory cascades [36,37]. Under the present experimental conditions, H_2_O_2_ treatment failed to induce a significant elevation of IL-6 and IL-8 levels. Notably, compared with the control and H_2_O_2_-treated groups, AKG supplementation demonstrated suppressive effects on the protein expression levels of both cytokines. This phenomenon may be attributed to the prolonged exposure time to H_2_O_2_, given that IL-6 and IL-8 typically function during the initial stages of cellular infection or injury. While interleukins contribute to immune surveillance under physiological conditions, their pathological overexpression can exacerbate inflammatory responses. The observed reduction in IL-6 and IL-8 expression by AKG potentially occurs through mitochondrial function improvement and oxidative stress mitigation, suggesting indirect regulatory mechanisms. Collectively, these findings indicate the existence of direct or indirect associations between AKG and interleukin regulation, though detailed mechanistic pathways require further experimental elucidation.

MDA, a hallmark product of lipid peroxidation, serves as a reliable indicator of oxidative stress intensity [38]. In murine colitis models, AKG has demonstrated efficacy in attenuating dextran sulfate sodium (DSS)-induced elevations of both MDA and H_2_O_2_ [39]. Our experimental findings align with these observations, revealing that H_2_O_2_ exposure significantly increased MDA levels, whereas AKG pretreatment induced a marked reduction in MDA content. Concomitantly, AKG administration substantially enhanced critical antioxidant parameters in oxidative stress-induced DPCs, including the GSH/GSSG ratio, SOD activity, and T-AOC. Glutathione, a pivotal endogenous antioxidant, derives its biosynthesis from glutamine, which serves as both a metabolic precursor and regulatory factor in GSH synthesis [40]. We hypothesize that AKG may augment intracellular GSH levels through the inhibition of glutamine catabolism, thereby promoting glutamine accumulation and subsequent GSH biosynthesis. Notably, SOD represents the sole enzymatic system capable of direct free radical neutralization, catalyzing the dismutation of superoxide radicals into molecular oxygen and hydrogen peroxide with exceptional specificity and catalytic efficiency [41]. While no direct evidence has established a mechanistic link between AKG and SOD activation, we postulate that the AKG-mediated enhancement of energy metabolism and oxidative damage mitigation might indirectly preserve SOD activity. This synergistic interaction between AKG’s metabolic support and SOD’s radical-scavenging function potentially establishes a comprehensive antioxidant defense network. Our results corroborate previous findings demonstrating AKG’s protective effects against H_2_O_2_-induced matrix degradation and apoptosis in chondrocytes, characterized by reduced ROS and MDA levels alongside elevated SOD activity and GSH/GSSG ratios [42]. This consistency across experimental models strongly suggests that AKG exerts a partial restoration of antioxidant enzyme activities compromised by H_2_O_2_ exposure, ultimately enhancing cellular antioxidant defenses through multifaceted mechanisms.

However, the molecular mechanisms by which AKG alleviates H_2_O_2_-mediated oxidative stress remain incompletely understood. To investigate these mechanisms, we systematically analyzed the expression levels of key regulatory genes. Compared to the control group, H_2_O_2_ exposure significantly upregulated *Nrf2* and *NQO1* expression, a trend further amplified by AKG cotreatment. The Nrf2-Keap1 axis constitutes a highly conserved cellular defense system against oxidative stress. Under physiological conditions, Nrf2 is sequestered in the cytoplasm through binding to Keap1, marking it for proteasomal degradation. Under oxidative stress, however, Nrf2 dissociates from Keap1, undergoes nuclear translocation, and forms a heterodimer with sMaf proteins to activate the transcription of antioxidant response element (ARE)-driven genes [43]. As a critical component of cellular adaptive responses, *NQO1* expression is directly regulated by the Nrf2 pathway [44]. This is consistent with previous studies demonstrating that sublethal H_2_O_2_ doses activate Nrf2 signaling as a hormetic stimulus to enhance antioxidant capacity [45,46,47]. Our findings suggest that AKG potentiates Nrf2 pathway activation under H_2_O_2_-induced oxidative stress. The concomitant upregulation of antioxidant genes further supports this conclusion. Intriguingly, divergent expression patterns were observed for *GPX4* and *Cas8*. AKG treatment reduced *GPX4* mRNA levels while increasing *Cas8* expression. We hypothesize that AKG-induced Nrf2 activation may accelerate *GPX4* protein synthesis, leading to the transient depletion of cytoplasmic *GPX4* mRNA pools due to enhanced transcription–translation coupling. Conversely, elevated *Cas8* expression likely reflects the activation of the extrinsic apoptotic pathway via H_2_O_2_/AKG-mediated JNK/MAPK signaling, though further validation is required.

Western blot analysis revealed that AKG treatment reduced cytoplasmic Nrf2 levels while significantly increasing those of nuclear Nrf2 and phosphorylated Nrf2 (P-Nrf2). Phosphorylation, a critical post-translational modification of Nrf2, regulates its stability, nuclear translocation, DNA binding affinity, and nuclear export [48]. The observed nuclear accumulation and phosphorylation of Nrf2 indicate that AKG enhances Nrf2 transcriptional activity by facilitating nuclear translocation and stabilizing its interaction with ARE sequences, thereby amplifying antioxidant gene expression. Building on earlier evidence of AKG’s modulation of MAPK signaling, we investigated a potential regulatory interplay between the MAPK and Nrf2 pathways. Although AKG differentially modulated the phosphorylation levels of MAPK proteins (ERK1/2, JNK, and p38) under oxidative stress, only ERK inhibition significantly suppressed Nrf2 expression. This aligns with studies demonstrating the ERK/Nrf2-dependent mitigation of metabolic stress in murine hyperlipidemia models and ERK/p38-mediated Nrf2 activation in astrocytes [49,50]. Notably, in t-BHP-induced hepatocyte oxidative stress, andrographolide-activated Nrf2 nuclear translocation was attenuated by ERK/Akt inhibitors, paralleling our observations [51]. Collectively, these findings support the existence of an AKG-ERK-Nrf2 regulatory axis under H_2_O_2_-induced oxidative stress. Cotreatment with an ERK1/2 inhibitor abolished AKG’s protective effects, as evidenced by restored ROS levels and reduced cell viability compared to AKG-treated groups. This complete reversal confirms ERK signaling as the central hub mediating AKG antioxidant activity. Our study provides novel mechanistic insights into AKG’s cytoprotective role, highlighting its dual regulation of the MAPK and Nrf2 pathways to counteract oxidative damage. However, this study has certain limitations. For example, we cannot completely rule out the oxidative decarboxylation of AKG by H_2_O_2_, and a further validation of the downstream NRF2 signaling pathway is still lacking.

## 5. Conclusions

In conclusion, our study demonstrates that AKG alleviates H_2_O_2_-induced oxidative damage in DPCs by reducing the levels of inflammatory cytokines, enhancing antioxidant capacity, and improving anti-apoptotic ability. This protective effect is likely mediated through the ERK/Nrf2 signaling pathway. While this investigation presents novel findings, certain limitations should be acknowledged. Future studies should further validate the role of Nrf2 and its downstream targets in the AKG-mediated enhancement of antioxidant capacity through either Nrf2 knockout or overexpression approaches. Collectively, this research provides the first elucidation of the molecular mechanism by which the endogenous metabolite AKG mitigates H_2_O_2_-mediated oxidative injury. These findings not only highlight AKG’s therapeutic potential in treating oxidative stress-related disorders but also broaden our understanding of its functional role in the antioxidant defense system.

## Figures and Tables

**Table 1:** Primer sequences.

Gene	Sequences (5′→3′)	Accession No.	Product Size/bp
*Keap1*	F: CCTCAACCGCCTGCTCTATGC R: ATCCGCCACTCGTTCCTCTCC	XM_008251549.4	96
*Nrf2*	F: AAGCAACTCAGCACCTTGTATCTGG R: GAATACATTGCCGTCCCTCGTCTG	XM_051849400.2	114
*NQO1*	F: AGCGGCTCCATGTACTCTCTCC R: GGAGTGTGCCCGATGCTGTATG	XM_070062671.1	137
*Cas3*	F: CTAAGCCACGGTGATGAAGGAGTC R: CACTGTCTGTCTCGATGCCACTG	NM_001354777.2	175
*Cas8*	F: CTGTCCAGGGGAGCCAGTGAG R: GCGGTGTCTGGGCATTTCTCTC	XM_051849294.2	136
*ALP*	F: TGCACAGAGCAAGAGAAGGA R: TCTCCCAGGAACAGGATGAC	XM_017346489.1	146
*GPX4*	F: CAGGAGAACGCCAAGAATGAGGAG R: GTTCACCTCGCACTTCTGGAAGAG	NM_001085444.1	105
*SOD*	F: TTTCTGGACAAACCTGAGCCCTAAC R: CCGTCAGCCTCTCCTTGAACTTG	XM_051854201.1	110
*CAT*	F: CAGCCAGCGACCAGATGAAGAAG R: CTGCCGTGATGATGTTCAGTTTGTC	XM_002709045.4	114
*Bcl2*	F: GTTCGGTGGGGTCATGTGTGTG R: AGGTGCCGGTTCAGGTACTCAG	XM_008261439.3	99
*Bax*	F: TATGGGCTGGACGCTGGACTTC R: AGATGGTGAGTGAGGCGGTGAG	XM_002723696.4	155

## Data Availability

The experimental data presented in this study can be requested from the corresponding author.
